# Fermentation-induced modifications to the structural, surface, and functional properties of quinoa proteins

**DOI:** 10.1007/s10068-025-01930-y

**Published:** 2025-07-05

**Authors:** Mohammad Alrosan, Motasem Al-Massad, Hadeel J. Obeidat, Sofyan Maghaydah, Muhammad H. Alu’datt, Thuan-Chew Tan, Zhengrui Liao, Omar Alboqai, Mohammad Ebqa’ai, Mohammed Ali Dheyab, Ali Madi Almajwal

**Affiliations:** 1 Department of Food Science and Nutrition, Faculty of Agriculture, Jerash University, P.O. Box 311, Jerash, 26150 Jordan; 2 QU Health, College of Health Sciences, Qatar University, P.O. Box 2713, Doha, Qatar; 3 Department of Animal Production and Protection, Faculty of Agriculture, Jerash University, Jerash, 26250 Jordan; 4 Department of Nutrition and Food Technology, Faculty of Agriculture, Jordan University of Science and Technology, P.O. Box 3030, Irbid, 22110 Jordan; 5 Department of Food Science & Nutrition, College of Life Sciences, Kuwait University, P.O. Box. 5969, 13060 Safat, Kuwait; 6 Food Technology Division, School of Industrial Technology, Universiti Sains Malaysia, 11800 USM Penang, Malaysia; 7 Department of Chemistry, Physics, and Engineering, Cameron University, Lawton, OK 73505 USA; 8 School of Physics, Universiti Sains Malaysia, 11800 USM Penang, Malaysia; 9 Department of Community Health Sciences, College of Applied Medical Sciences, King Saud University, P.O. Box 10219, 11433 Riyadh, Saudi Arabia

**Keywords:** Quinoa proteins, Fermentation, Protein quality, Solubility, Digestibility

## Abstract

This research study investigates the relationship between the structural characteristics, water solubility, and protein digestibility of quinoa proteins (QPs) during *Lactiplantibacillus plantarum* fermentation. The fermentation process induces structural modifications in QPs, thereby enhancing their surface properties and functional attributes. Using advanced analytical techniques, such as ultraviolet, fluorescence, and FT-IR spectra, it has been demonstrated that fermented QPs exhibit significant structural changes (P < 0.05) compared to unfermented QPs. Notably, fermentation significantly increases the digestibility of QPs from 78.13 to 85.24%, thereby enhancing their nutritional value. Furthermore, surface properties undergo modifications, with a decrease in surface charge from −32.82 to −39.63 and a reduction in surface hydrophobicity from 580 to 382 a.u. These findings underscore the transformative impact of *L. plantarum* fermentation on QPs, resulting in improved digestibility, modifications in protein structure, and enhanced nutritional benefits. Further research on water kefir-based fermentation is needed to optimize its quality.

## Introduction

The extensive consumption of meat presents substantial challenges to both the environment and human health, necessitating urgent advocacy for sustainable, plant-based alternatives to meat (Alrosan et al., 2022). Plant-based foods are abundant in diverse bioactive compounds, including antioxidants, polyphenols, flavonoids, and saponins (Alrosan et al., 2024). These bioactive compounds play a pivotal role in conferring health benefits to consumers.

Quinoa proteins (QPs) are renowned for their distinctive nutritional profile (Alrosan et al., 2022), which encompasses a high content of essential amino acids. The elucidation of the structural, surface, and functional properties of these proteins is pivotal in comprehending fermentation-induced modifications (Alrosan et al., 2023a; Cui et al., 2023; Al‐Qaisi et al., 2024). The amino acids profile of QPs is characterized by a harmonious balance of essential and non-essential amino acids (Cui et al., 2023). In recent years, fermentation has been explored as a methodology to augment the bioavailability and digestibility of these proteins, potentially leading to enhanced health outcomes (Al‐Qaisi et al., 2024). The digestibility of proteins is influenced by a multitude of factors, including the fermentation process, which can modify protein structure composition, surface charge, surface hydrophobicity, and secondary and tertiary structures (Al‐Qaisi et al., 2024; Luo et al., 2024; Hurtado-Murillo et al., 2025; Zheng et al., 2025). The modifications of the protein structure that impact the functional properties of plant-based proteins, such as water solubility (Zheng et al., 2025; de Amarante et al., 2025), emulsion formation (Zheng et al., 2025), and protein digestibility (Xu et al., 2021; Alrosan et al., 2024), are of particular significance.

Current practical applications of QPs are limited due to consumer perceptions of their sensory attributes and functional properties. Notably, QPs exhibit approximately 76.6% water solubility (de Amarante et al., 2025) and 76.4% protein digestibility (Alrosan et al., 2023a). The water solubility of proteins significantly influences their functional properties, with high-quality proteins being easily digestible and providing a comprehensive range of essential amino acids (Xu et al., 2021).

Lactic acid bacteria (LAB) play a crucial role in the fermentation process of QPs, modifying their structural and functional properties (Alrosan et al., 2024; Hurtado-Murillo et al., 2025). The interaction between LAB and QPs leads to enhanced solubility (Alrosan et al., 2024; Hurtado-Murillo et al., 2025), improved emulsifying capacity (Nie et al., 2022), and enhanced nutritional value (Alrosan et al., 2024). Al‐Qaisi et al. (2024) reported a notable increase in the digestibility of pea proteins from 88 to 94% due to structural modifications attributed to the activity of LAB and their enzymes. Furthermore, Li et al. (2024) demonstrated that the water solubility of egg yolk increased from 21.26 to 32.81 g/100 g following treatment with fermentation lactic acid bacteria, resulting in changes in surface properties and secondary protein components.

The structural characteristics of plant-based proteins, as elucidated in the reports by Xu et al. (2021) and Alrosan et al. (2024), revealed a substantial rigid configuration that significantly impacts their digestion process in the gastrointestinal tract. Consequently, this research study aims to explore the intricate relationship between the protein structures of QPs, their water solubility, and the extent of protein digestibility that occurs during the fermentation process involving *Lactiplantibacillus plantarum.* Additionally, the study will investigate the impact of *L. plantarum* on the surface properties of QPs.

## Materials and methods

The quinoa grains (*Chenopodium quinoa Willd*.) were obtained from a local market in Jordan. QPs were prepared from these quinoa grains using alkaline extraction at pH 9.0 to solubilize the proteins. Subsequently, isoelectric precipitation at a pH of 5.0 was employed to facilitate the precipitation of QPs, followed by further purification before lyophilization and milling to produce fine lyophilized QPs powder (Alrosan et al., 2023a). The standards for caffeic acid, rutin, chlorogenic acid, catechin, ferulic acid, quercetin, epicatechin, sinapic acid, gallic acid, sinapic, and syringic acid were obtained from Sigma-Aldrich Inc (St. Louis, USA). The supplementary materials were obtained from the research laboratory (Pharmaceutical Research Center, Irbid, Jordan), handled according to the manufacturer’s guidelines, and stored at around 4 °C.

### Preparation of fermented QPs

The specific LAB strain used was *Lactiplantibacillus plantarum*, which was cultured in Man Rogosa Sharpe (MRS) broth. The bacteria were cultivated at 37 °C, which is a standard temperature for the growth of LAB. The fermented solution of QPs was prepared using deionized water at a ratio of 1:11 (w/v), inoculated with *Lactiplantibacillus plantarum* at a concentration of 10⁷ log CFU/g, and incubated at 35 °C, with samples collected every 6 h.

### Enumeration of microorganisms and pH determination

Microorganisms were enumerated during fermentation using MRS. Serial dilutions (10^1^ to 10⁹) were prepared on the samples to ensure acceptable colony counts (30 to 300 colonies). The plates were incubated at 37 °C for 48 h to facilitate colony development, including slower-growing microorganisms. The pH of the samples was determined using a calibrated pH meter.

### Water solubility

The water solubility of fermented QPs was evaluated according to the method outlined by Alrosan et al. (2024). Homogenized mixtures of QPs (0.2 g) and distilled water were adjusted to a pH of 7.0 using 0.2 M NaOH or HCl. Subsequently, the mixtures were stirred at 1,000 rpm for 60 min using a magnetic stirrer, with distilled water added to adjust the concentration to 1% (w/v) before completion. The nitrogen content in the whole sample (*W*_S_), supernatant (*S*_N_), and blank (*B*_S_) was determined using the Kjeldahl method (AOAC 930.29) to calculate the water-soluble nitrogen percentage relative to the total nitrogen.

$$\text{Water solubility} \left(\%\right)= \frac{\left({\text{B}}_{\text{N}}-{\text{B}}_{\text{s}}\right)}{{\text{W}}_{\text{s}}}\times 100\%$$

### In vitro protein digestibility (IVPD)

The protein digestibility of unfermented and fermented QPs was evaluated according to Al‐Qaisi et al. (2024). Protein samples (250 mg) were dissolved in a pepsin-HCl solution and heated at 37 °C for 3 h. Sodium azide was added to prevent microbial growth, followed by the addition of NaOH, phosphate buffer, and pancreatin. The mixtures were incubated at 37 °C for 24 h. After centrifugation at 10,000 ×*g* for 20 min, the nitrogen content in the whole sample (*N*_W_), supernatant (*N*_S_), and blank (*N*_B_) was determined using the Kjeldahl method (AOAC 930.29). Protein digestibility was calculated using the following formula.

$${\text{IVPD}} \left(\%\right)= \frac{\left({\text{N}}_{\text{S}}-{\text{N}}_{\text{B}}\right)}{{\text{N}}_{\text{W}}}\times 100\%$$

### Surface hydrophobicity (H_0_)

Surface hydrophobicity was assessed using a fluorescence spectrophotometer (Agilent Cary Eclipse) based on the procedure outlined by Al-Qaisi et al. (2024). Protein samples (100 mg) were homogenized with sodium phosphate and stirred for 2 h. A calibration curve was created to establish a linear relationship between fluorescence intensity (FI) to protein concentration, spanning a range of protein concentrations from 0.1 to 0.01% (w/v). ANS (8 mM) was added to probe the hydrophobic regions of proteins. The samples were vortexed, allowed to remain in the dark for 15 min, and subsequently analyzed at excitation and emission wavelengths of 390 and 470 nm, respectively. Fluorescence intensity was measured and corrected by subtracting the blank sample. Linear regression analysis was employed to determine the correlation between FI and protein concentration, with the slope representing the change in FI with protein concentration.

### Zeta potential

The surface charge of both fermented and unfermented QPs was determined using a Zeta potential analyzer (Malvern Panalytical, Mastersizer 2000). The analysis was conducted at a 0.05% (w/v) concentration of fermented QPs, following the procedure outlined by Al-Qaisi et al. (2024). The refractive indices of the protein samples (1.450) and the dispersant (distilled water, 1.330) were measured to determine the Zeta potential, which represents the electric charge of the surface of particles suspended in a liquid.

### Fourier-transform infrared (FTIR) spectroscopy

The analysis was conducted using a FTIR spectrometer, with spectral measurements spanning the range of 400 and 4000 cm⁻^1^. The amide I band in the FTIR spectrum, which corresponds to the stretching vibrations of the C=O bond in the amide linkage, exhibits sensitivity to the secondary structure of proteins. By analyzing this band, it is possible to gain insights into the protein’s conformation. The methodology employed for identifying the amide I group adhered to the procedure outlined by Alrosan et al. (2021).

### Spectrofluorometry

The protein samples were diluted with distilled water to a concentration of 0.001% (w/v) and analyzed using fluorescence spectroscopy to investigate their fluorescence properties. This technique is sensitive to fluorescence emissions, which can indicate structural or conformational changes in proteins, such as those occurring during fermentation. Intrinsic fluorescence, emitted by aromatic amino acids including tryptophan, was measured using an excitation wavelength of 280 nm, corresponding to the absorption maximum of tryptophan. The Cary Eclipse fluorescence spectrophotometer was utilized, with excitation and emission bandwidths set to 10 nm.

### UV-spectrometry

A UV spectrophotometer (Shimadzu, UV-3600, Kyoto, Japan) was employed to analyze both fermented and unfermented QPs. The spectra were recorded in the ultraviolet range, spanning from 190 to 350 nm, to ascertain the absorption of ultraviolet light by the samples. This analysis elucidates the structural and compositional characteristics of the QPs.

### Phenolic compounds

Phenolic compounds were analyzed according to the method described by Al-Qaisi et al. (2024). The protein samples were dissolved in methanol at a 1:8 ratio and sonicated at 35 °C for 3 min. Subsequently, the mixtures were stored at 4 °C and brought to room temperature until the residues turned white. The mixtures were centrifuged at 10,000 ×*g* for 15 min to collect the supernatants, which were then filtered before HPLC analysis. Separations were achieved using a reverse-phase C18 column, with hydrophobic interactions as the basis for separation. The mobile phases were acetonitrile (A) and 1% acetic acid in distilled water (B). A gradient profile was employed to separate the phenolic compounds, which were detected at wavelengths of 254 and 272 nm.

### Total phenolic content (TPC)

The TPC of protein samples during fermentation was determined using the UV-3600 UV–vis spectrophotometer according to the method described by Al-Qaisi et al. (2024). Mixtures of protein samples (100 µL), Folin-Ciocalteu reagent (500 µL), and distilled water (8.4 mL) were vortexed for 4 min. Then, 5% sodium carbonate solution was added, and the mixtures were vortexed again for 4 min. The solution was incubated in the dark for 1 h, and absorption was measured at 725 nm. TPC was calculated as mg of gallic acid equivalent per 100 g (mg GAE/100 g) using a calibration curve based on the identified gallic acid concentrations.

### Statistical analysis

Statistical analysis was conducted using SPSS version 23.0 for efficient data management and analysis. Duncan’s multiple range test was employed to compare the means between different groups, while one-way ANOVA was utilized to assess the overall differences among multiple groups. A statistical significance level of *P* < 0.05 was established for the analysis.

## Results and discussion

### Water solubility of fermented QPs

The water solubility of plant-based proteins is a crucial characteristic that significantly influences their functional properties (Maghaydah et al., 2025; Xu et al., 2021; Zheng et al., 2025). In particular, the solubility of QPs can be notably enhanced through fermentation, resulting in increased availability for various applications in food science and nutrition (Alrosan et al., 2023b; de Amarante et al., 2025; Hurtado-Murillo et al., 2025). The solubility of QPs throughout the fermentation process, as depicted in Table 1, is presented. The solubility of QPs throughout the fermentation process was approximately 78.32%, which significantly (*P* < 0.05) increased to 85.27% after 24 h of fermentation. This substantial increase in solubility suggests that the fermentation process facilitates the breakdown of QPs into smaller protein molecules, leading to an enhancement of soluble protein content.

**Table 1:** Changes in the pH, total soluble solids (TSS, °Brix), protein digestibility (%), and sugar profile (g/L) of unfermented (Hour 0) and water quinoa proteins

Parameters	Fermentation period (Hour)					*P* value
	0	6	12	18	24	
Solubility	78.32 ± 0.8^c^	81.26 ± 1.0^b^	82.66 ± 2.66^ab^	83.96 ± 1.72^ab^	85.27 ± 1.06^a^	< 0.05
pH	6.81 ± 0.01^§^	5.75 ± 0.02^b^	4.23 ± 0.01^c^	3.93 ± 0.02^d^	3.80 ± 0.01^e^	< 0.05
Protein digestibility	78.13 ± 1.10^d^	80.07 ± 1.0^c^	81.94 ± 0.81^bc^	83.17 ± 0.7^b^	85.24 ± 1.52^a^	< 0.05
Surface hydrophobicity	580 ± 9.0^a^	498 ± 10.5^b^	446.6 ± 7.0^c^	411 ± 9.5^d^	382 ± 12^e^	< 0.05
Surface charge	−32.83 ± 1.10^d^	−34.53 ± 0.93^c^	−36.3 ± 0.21^c^	−37.63 ± 0.70^b^	−39.63 ± 1.06^a^	< 0.05
L.* plantarum*	5.92 ± 0.02^e^	6.93 ± 0.02^d^	7.39 ± 0.07^c^	8.37 ± 0.05^b^	8.94 ± 0.03^a^	< 0.05

The data presented in the table represent the mean ± standard deviation (n = 3). Values with different superscripts within the same row are statistically significant from each other (*P* < 0.05)

A study conducted by Hurtado-Murillo et al. (2025) demonstrated that the water solubility of a quinoa and chickpea protein blend (90:10) increased by approximately 5.2 to 32% after 24 h of fermentation with *Lactobacillus* species. These findings align with the observations reported by Liu et al. (2023a) and Liu et al. (2023b), which indicated an improvement in the percentage of water solubility in chickpeas after fermentation by *Lactiplantibacillus plantarum*.

During fermentation, certain microorganisms, such as *Lactobacillus acidophilus*, produce organic acids, including lactic acid and acetic acid (Ayala-Niño et al., 2019; Shi et al., 2021; Alrosan et al., 2023a; Liu et al., 2023a; Maghaydah et al., 2025). These organic acids contribute to the acidification of the fermentation solution. A study by Shi et al. (2021) proposed that structural modifications in globular proteins can lead to alterations in the protein arrangement of the pea proteins. These modifications encompass alterations to the primary, secondary, tertiary, or quaternary structures of the pea proteins. The utilization of *Bacillus subtilis* in the fermentation process resulted in a substantial increase in the quantity of soluble proteins found in the cottonseed meals (Sun et al., 2015). Soluble proteins are characterized by their rapid dissolution or dispersion in water. Furthermore, fermentation, in addition to hydrolysis, is the process of breaking down larger protein molecules into smaller peptides and amino acids (Alrosan et al., 2023b; Hurtado-Murillo et al., 2025). Consequently, fermentation is considered effective in breaking down QPs, leading to an enhancement in protein quality. This process of breakdown facilitates the solubility of the proteins.

### pH solution of QPs

The fermentation processes are catalyzed by enzymes produced by microorganisms such as lactic and acetic acids fermenting bacteria (Shi et al., 2021). Enzyme activity is often pH-dependent, and maintaining an optimal acidity level is essential for ensuring the effectiveness of these enzymes in breaking down proteins into desirable components (Alrosan et al., 2023a; Liu et al., 2023b; Maghaydah et al., 2025; Shi et al., 2021;). The pH of the fermented QP solution during the fermentation period (Table 1) exhibited a significant (*P* < 0.05) decline, decreasing from an initial value of 6.81 ± 0.01 to 3.80 ± 0.01 within 24 h. LAB have been the subject of extensive research in food fermentation due to their ability to metabolize carbon sources such as glucose and fructose into lactic acid. The resulting microbiological acidification, characterized by low pH (< 4.5), high acidity (Hurtado-Murillo et al., 2025), and the activation of extracellular microbial proteases produced during fermentation, plays a crucial role in releasing peptides from plant-based proteins, thereby enhancing protein hydrolysis and directly improving protein properties. Various microorganisms, including *Streptococcus* spp., *Bacillus* spp., *Bifidobacterium* spp., and *Lactobacillus* spp., are commonly utilized as probiotics. For instance, *Lactobacillus acidophilus* is one of the most well-known LAB species. It thrives optimally at temperatures of 35–42 °C and pH levels of approximately 5–6. As a homofermentative bacterium, *Lactobacillus acidophilus* converts carbohydrates into lactic acid and carbon dioxide. Furthermore, it exhibits remarkable fermentation efficiency and can grow in plant-based substrates such as cereals and legumes (Al‐Qaisi et al., 2024; Liu et al., 2023a; Liu et al., 2023b; Nie et al., 2022; Maghaydah et al., 2025).

### Digestibility of QPs

The digestibility of fermented proteins is a crucial factor to consider when evaluating their nutritional value and potential health benefits (Cui et al., 2023). Digestibility refers to the body's capacity to absorb and utilize protein and its components, including amino acids and peptides (Galani et al., 2022; Maghaydah et al., 2025). The digestibility of QPs throughout fermentation (Table 1) and the digestibility of the control sample (unfermented QPs) approximately 78.13%, are primarily attributed to the presence of antinutritional factors, such as saponins and phytic acid (Alrosan et al., 2023a, b), which inhibit protein absorption. Furthermore, the structural characteristics of QPs, including their solubility and molecular weight, contribute to reduced digestibility (Al‐Qaisi et al., 2024). As presented in Table 1, the digestibility of fermented QPs significantly increased to 85.24% following 24 h of fermentation. The acidity generated during fermentation may alter protein conformation, enhancing the accessibility of QPs to proteolytic enzymes. This process facilitates the breakdown of proteins into smaller peptides and free amino acids, thereby increasing the protein digestibility of QPs.

The presence of antinutritional factors, such as tannins, saponins, and other phenolic compounds, in QPs can indeed affect protein digestibility (Shi et al., 2021; Alrosan et al., 2023b). This suggests a reduction in protein crosslinking (Alrosan et al., 2021; Maghaydah et al., 2025). Reducing protein crosslinking generally refers to breaking down or preventing the formation of strong chemical bonds between protein molecules, resulting in changes in the fermented protein structure. Therefore, the LAB in this study may break down protein (Alrosan et al., 2021, 2023b; Moretti et al., 2022). This process can enhance protein solubility (Hurtado-Murillo et al., 2025), digestibility (Alrosan et al., 2021; Maghaydah et al., 2025; Moretti et al., 2022), and produce protein hydrolysis throughout the fermentation period, resulting in the production of amino acids and peptides (smaller protein fragments) (Alrosan et al., 2023a). The findings of the study align with the well-established understanding of the proteolytic activity of LAB during fermentation by Alrosan et al. (2021) and Moretti et al. (2022). Notably, *Lactobacillus* spp. is known for its ability to hydrolyze proteins into smaller peptides, which can subsequently be broken down into amino acids through the activity of various intracellular peptidases, including proline-specific peptidases, aminopeptidases, and carboxypeptidases. This suggests that peptide bonds were effectively broken down.

### Surface hydrophobicity of fermented QPs

The pH level during fermentation undergoes alterations due to the production of organic acids. This pH variation significantly influences the charge distribution on protein surfaces, affecting their properties. For instance, at elevated pH levels, specific residues undergo deprotonation, resulting in a more negative charge distribution on the protein surface. This charge change modulates the protein's solubility, stability, and enzymatic activity. Consequently, pH plays a crucial role in regulating the behavior and functionality of surface proteins.

Conversely, the altered charge distribution can impact protein–protein interactions and binding affinities. The surface hydrophobicity of QPs (pre-fermentation) is approximately 580 ± 9.0 (Table 1). Environmental conditions, such as temperature variations, humidity changes, ionic strength alterations, and enzymatic processes, can influence the surface properties of quinoa proteins, including hydrophobicity (Al‐Qaisi et al., 2024; Liu et al., 2023a). Notably, during fermentation, the surface hydrophobicity of QPs significantly (*P* < 0.05) decreases and reaches approximately 382 ± 12 after 24 h.

Microorganisms, particularly certain LAB strains, are known to produce proteolytic enzymes during fermentation (Ayala-Niño et al., 2019; Maghaydah et al., 2025; Moretti et al., 2022). These enzymes can break down the peptide bonds in proteins, leading to the unfolding or denaturation of the protein structures (Alrosan et al., 2023b). Unfolding exposes hydrophilic regions of the proteins, thereby reducing the overall surface hydrophobicity. However, the process of protein unfolding is essential for the optimal functioning of proteins (Alrosan et al., 2023a). It enables them to adopt their specific structures, which determine their function and interactions with other molecules (Gantumur et al., 2023). Furthermore, the fermentation of whey proteins results in a decrease in surface hydrophobicity due to enhanced negative charge of the proteins and the exposure of hidden hydrophobic groups (Alrosan et al., 2023a).

### Surface charge of fermented QPs

The surface charge of proteins, typically expressed as the protein's net charge at a given pH, is a pivotal parameter that governs various aspects of protein behavior, including solubility, interactions with other molecules, and stability (Alrosan et al., 2023a). The surface charge of QPs (pre-fermentation) is approximately −32.83 ± 1.10 mV (Table 1). Notably, the surface charge of fermented QPs undergoes a significant increase (*P* < 0.05) to reach −39.63 ± 1.06 mV throughout the fermentation process. During fermentation, a consistent reduction (*P* < 0.05) in the surface charge of the fermented QPs is observed. A study by Zhang et al. (2020) demonstrated that augmenting the degradation of other compounds, such as carbohydrates, leads to an elevation in the surface charge of fermented proteins. The utilization of simple carbohydrates by fermenting microorganisms, including LAB, for their development and growth has resulted in an overall increase in the surface charge of the fermented proteins. This decrease in surface charge is attributed to the consumption of sugars by these LAB, which subsequently produces organic acids and other metabolites. These metabolites can alter the pH and ionic composition of the fermented proteins, thereby inducing an increase in their surface charge. This research findings align with previous investigations on protein fermentation, including whey (Zhang et al., 2020), chickpea (Liu et al., 2023b), pea (Moretti et al., 2022), and quinoa (Ayala-Niño et al., 2019).

### Protein structures QPs

#### Secondary protein structure

FT-IR spectroscopy is a sensitive technique capable of detecting molecular vibrations in proteins. It is a valuable tool for elucidating the conformational changes, structural features, and overall characteristics of diverse protein molecules. This study revealed the secondary protein conformation of QPs throughout the fermentation process (Table 2). The secondary protein structures of QPs, comprising α-helices, β-turns, random coil (RC), and β-sheets, were found to be 22.14, 22.26, 15.33, and 40.25%, respectively. Notably, these percentages exhibited significant variation (*P* < 0.05) during fermentation. The α-helical structure of fermented QPs exhibited a significant (*P* < 0.05) decrease, reaching 16.52% at the end of fermentation.

**Table 2:** Evaluation of the proportion of secondary protein components of quinoa proteins (QPs) during the fermentation

Secondary protein components	Peak (cm^−1^)	Time (hr)					*P*-value
		QPs	6	12	18	24	
β-Sheet							
	1,614	15.70 ± 0.10^c^	16.25 ± 0.07^b^	16.28 ± 0.16^b^	16.78 ± 16.78^a^	16.84 ± 0.03^a^	< 0.05
	1,622	14.34 ± 0.08^b^	14.42 ± 0.04^b^	14.50 ± 0.08^b^	14.80 ± 0.05^a^	14.95 ± 0.20^a^	< 0.05
	1,633	10.20 ± 0.05^d^	10.53 ± 0.08^c^	10.68 ± 0.10^b^	10.95 ± 0.05^a^	10.97 ± 0.07^a^	< 0.05
β-Sheet (Ʃ)		40.25 ± 0.20	41.21 ± 0.03	41.47 ± 0.30	41.54 ± 0.17	42.77 ± 0.20	
Random coils	1,645	15.33 ± 0.10^d^	15.84 ± 0.09^c^	16.13 ± 0.08^b^	16.53 ± 0.09^a^	16.60 ± 0.04^a^	< 0.05
α-Helix	1,654	22.14 ± 0.13^a^	20.40 ± 0.40^a^	18.97 ± 0.21^b^	16.93 ± 0.08^c^	16.52 ± 0.09^d^	< 0.05
β-Turn							
	1,668	5.88 ± 0.10^d^	6.02 ± 0.06^c^	6.23 ± 0.4^b^	6.39 ± 0.07a	6.42 ± 0.50^a^	< 0.05
	1,681	9.24 ± 0.06^c^	9.30 ± 0.04^c^	9.52 ± 0.05^b^	9.75 ± 0.04^a^	9.79 ± 0.07^a^	< 0.05
	1,693	7.13 ± 0.10^c^	7.20 ± 0.10^c^	7.66 ± 0.06^b^	7.83 ± 0.05^a^	7.87 ± 0.08^a^	< 0.05
β-Turn (Ʃ)		22.26 ± 0.10^d^	22.53 ± 0.10^c^	23.41 ± 0.08^b^	23.98 ± 0.17^a^	24.09 ± 0.80^a^	< 0.05
α-Helix: β-Sheet		55.01	49.49	45.76	39.80	38.63	

The data presented in the table represent the mean ± standard deviation (n = 3). Values with different superscripts within the same row are statistically significant from each other (*P* < 0.05)

Furthermore, as reported by Alrosan et al. (2021), a decrease in α-helix content has been observed to enhance the accessibility of proteolytic enzymes to the protein substrate, facilitating the breakdown and digestion process. Significant (*P* < 0.05) changes were also observed in the percentage of RC, β-sheet, and β-turns, indicating a dynamic transformation in protein structures. The percentage of RC notably increased, reaching 16.60%, suggesting a shift in the conformational characteristics of the proteins under the fermentation conditions. The observed increase in the proportion of β-sheets during fermentation aligns with previous findings related to protein digestibility (Liu et al., 2023b; Alrosan et al., 2021). Additionally, a substantial rise in β-sheet content was observed, culminating at 42.77%, indicating a reorganization of protein secondary structures during fermentation.

Previous research has demonstrated that an elevated presence of β-sheets can have a detrimental impact on the digestibility of proteins (Alrosan et al., 2023b). Concurrently, the percentage of β-turns exhibited a significant (*P* < 0.05) increase, reaching 24.09%, underscoring the intricate and diverse alterations occurring in the protein folding patterns. The concomitant rise in β-sheets suggests a potential structural modification in the proteins that might impede the enzymatic breakdown during digestion (Cui et al., 2023). The arrangement of β-sheets within plant-based proteins is a pivotal determinant of their solubility, thereby influencing their behavior in protein digestibility (Al‐Qaisi et al., 2024). Notably, a study conducted by Liu et al. (2023b) revealed that the percentage of secondary protein structure underwent alterations after 12 h of fermentation by LAB. This finding indicates a substantial (*P* < 0.05) influence of LAB on the secondary protein structure throughout fermentation. Past studies conducted by Shi et al. (2021) and Alrosan et al. (2021) have elucidated that LAB and their enzymes may modify the protein structure of protein molecules. These modifications can encompass alterations in functional groups, protein unfolding, or the fragmentation of the proteins into smaller components. Enzymes play a pivotal role in these modifications by catalyzing specific chemical reactions, resulting in the desired protein structure transformation.

LAB introduce alterations to the ionic environment, which, in turn, influence the secondary structures, such as α-helix and β-sheet formations, leading to the overall transformation of the protein’s tertiary structure (Meinlschmidt et al., 2016). In this study, the quality of QPs has improved after fermentation. Furthermore, a lower α-helix to β-sheet ratio indicates a shift in the protein’s secondary structure, favoring a conformation that is more conducive to increased protein digestibility (Alrosan et al., 2023a). The ratio of α-helix to β-sheet decreased from 55.01 to 38.63% throughout the fermentation. This change in protein structure suggests that fermentation has altered the QPs in a manner that makes them easier to digest. The decrease in the α-helix to β-sheet ratio may also contribute to improved nutritional value and bioavailability of the fermented QPs.

### Tertiary protein structure

**Fig. 1:**
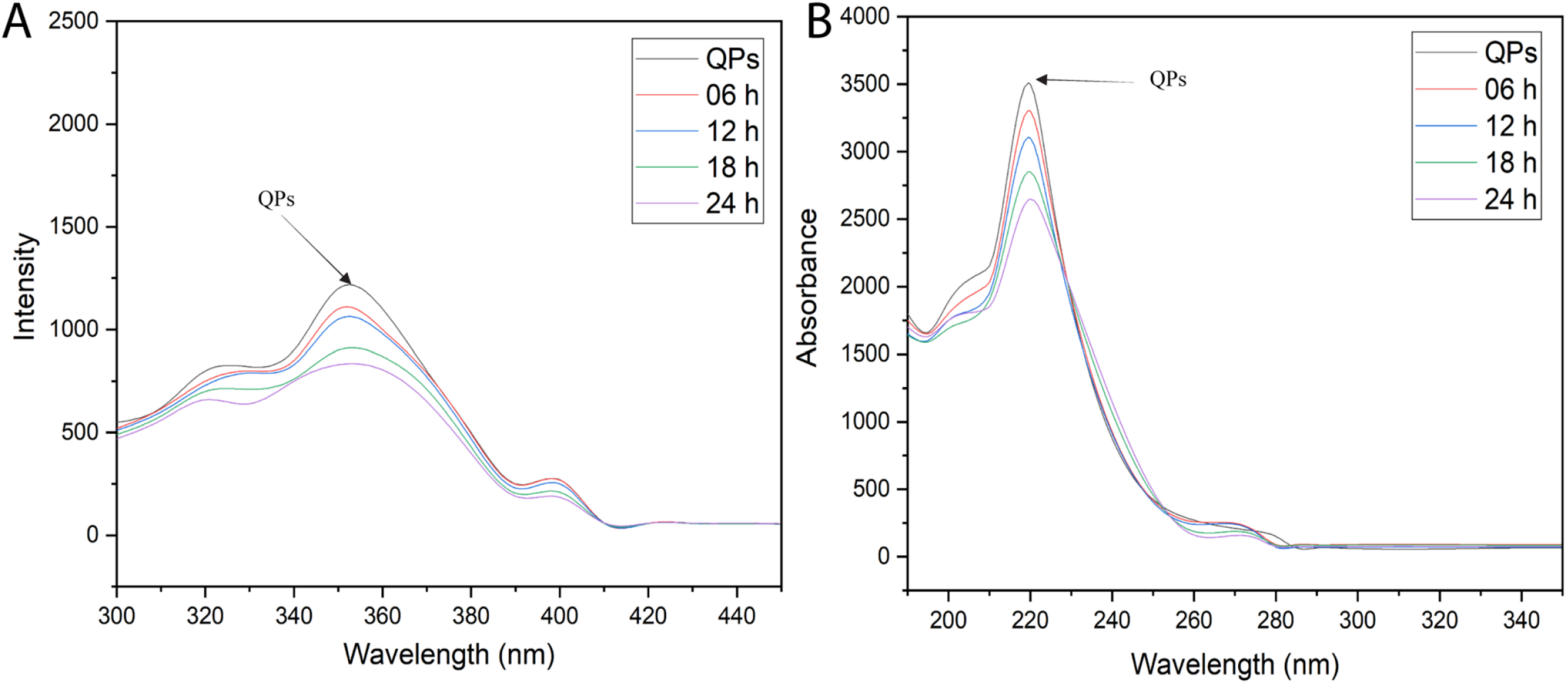
The fluorescence intensity and spectrophotometer of quinoa protein (1%, w/v) during the fermentation for 24 h. **A** The distribution of tryptophan residues in quinoa proteins using the ultraviolet spectra during the fermentation. **B** Fluorescence intensity of quinoa proteins using the fluorescence spectroscopy probe during the fermentation

Protein fluorescence spectra analysis offers a valuable tool for elucidating the structural organization of proteins within specific microenvironments. The fluorescence properties of these amino acids are highly sensitive to the microenvironment in which they are situated within the protein (Alrosan et al., 2023a). This method can be instrumental in examining proteins in their native state without the necessity of any external compounds. Effectively demonstrating the alteration of the protein structure, particularly the tertiary structure, during fermentation can be achieved by stimulating the amino acid tryptophan residues at a wavelength of 280 nm (Alrosan et al., 2024; Maghaydah et al., 2025). The predominant wavelength for the most intense fluorescence peak of tryptophan residues in hydrophobic protein environments is below 330 nm. The peak is believed to stimulate the indole ring of tryptophan, which exhibits exceptional sensitivity to changes in its surroundings. During the fermentation process of QPs, conformational changes can be observed by analyzing the fluorescence intensity and wavelength (Fig. 1A). The findings suggest that tryptophan may undergo alterations irrespective of variations in the pH of the fermenting solution. These alterations can be quantified by evaluating the protein binding level (Alrosan et al., 2023b). The intensity of the fluorescence spectrum gradually decreases during the fermentation. The results are determined by evaluating the level of binding of the internal proteins exhibited. Fermentation causes a gradual reduction in the intensity of the fluorescence spectra during the fermentation (Table 2). The modification of fluorescence intensity is widely recognized as an indicator of changes in the tertiary structure of proteins (Alrosan et al., 2021; Maghaydah et al., 2025).

### Conformation of protein structures

The absorption of UV light by chromophores is influenced by their local environment within the protein structure. Changes in the protein’s conformation, denaturation, or interactions with other molecules can affect the UV absorption spectra. Researchers utilize UV spectroscopy to study these changes and gain insights into the structure and behavior of proteins (Yang et al., 2021). Figure 1B illustrates the structure of QPs during the fermentation process. The UV peak absorbance values have been frequently employed for protein quantification and quality measurement. Furthermore, alterations in the maximum peaks can indicate modifications due to protein structure or denaturation. The decrease in the maximum intensity (approximately 220 nm) is associated with the peptide bond within the proteins and the phenylalanine residues contributing to the formation of the absorption peak (approximately 260 nm) (Yang et al., 2021).

The absorbance peak at 275 nm exhibited a consistent increase during fermentation, suggesting that the presence of double-bonded chains in tyrosine and tryptophan sequences caused the wavelength exposure of these amino acids in fermented QPs to increase. Additionally, this observation implies that the boost in absorbance is indicative of an increase in the concentration of tyrosine and tryptophan residues in the QPs during fermentation. These findings can potentially influence the nutritional value and functional properties of fermented QP products.

#### Phenolic compounds

**Fig. 2:**
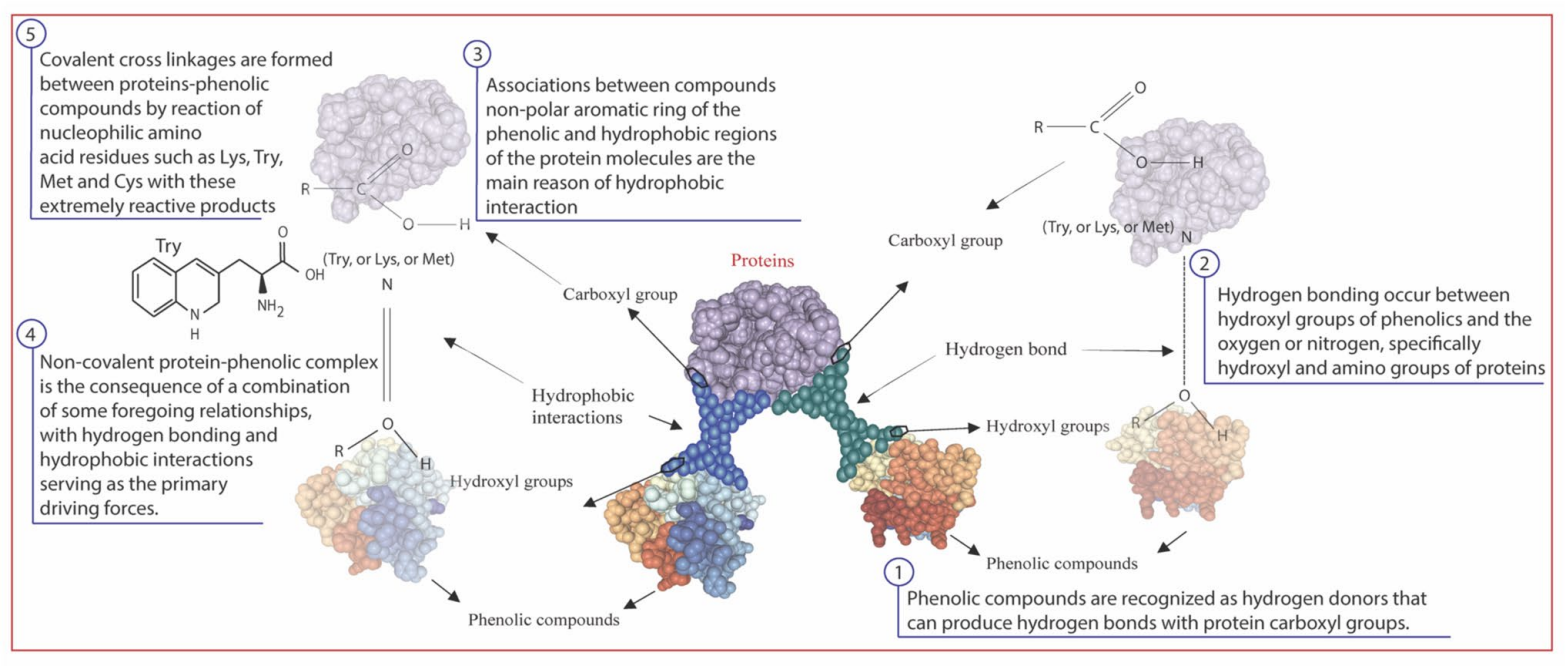
Proposed mechanism on the linkages between phenolic compounds and quinoa proteins during fermentation for soluble protein production

Current research elucidates the presence of various phenolic compounds within QPs during fermentation. Phenolic compounds can form complexes with proteins through interactions such as hydrogen bonding and hydrophobic interactions. These interactions can lead to alterations in protein conformation and function (Fig. 2). The total phenolic content in the QPs (pre-fermentation) is approximately 486.66 mg GAE/100 g, and it significantly (*P* < 0.05) increased to reach 651.60 mg GAE/100 g after 24-h of fermentation (Table 3). The observed increase in TPC after fermentation indicates that the fermentation process has resulted in modifications in the composition of phenolic compounds in the fermented QP samples. However, these increases can be attributed to the metabolic activity occurring during fermentation, which can hydrolyze the bonds between the substances (including proteins, carbohydrates, and phenolic compounds), thereby releasing soluble phenolic compounds. The findings presented in Table 3 suggest potential benefits and enhanced nutritional value associated with fermented quinoa protein samples.

**Table 3:** Changes in the total phenolic content (TPC, mg GAE/100 g) and phenolic compounds (mg/100 g) of quinoa protein throughout fermentation

Parameters	Fermentation period					*P* value
	0	6	12	18	24	
TPC	486.66 ± 6.0^e^	545.00 ± 7.0^d^	612.30 ± 6.0^c^	631.30 ± 5.0^b^	651.60 ± 14.0^a^	< 0.05
Catechin	29.37 ± 0.40^e^	31.23 ± 0.15^d^	31.93 ± 0.50^c^	33.57 ± 0.50^b^	34.43 ± 0.12^a^	< 0.05
Chlorogenic acid	53.07 ± 2.67^c^	57.03 ± 1.70^b^	58.50 ± 2.50^b^	62.60 ± 2.70^a^	63.13 ± 2.20^a^	< 0.05
Epicatechin	98.63 ± 1.42^d^	102.13 ± 1.0^c^	105.57 ± 1.50^b^	112.43 ± 1.90^a^	114.10 ± 2.00^a^	< 0.05
Quercetin	14.47 ± 1.35^e^	15.27 ± 1.50^d^	16.67 ± 1.21^c^	17.77 ± 1.60^b^	19.23 ± 1.50^a^	< 0.05
Rutin	5.63 ± 0.15^d^	5.80 ± 0.10^d^	6.23 ± 0.15^c^	6.89 ± 0.10^b^	7.10 ± 0.10^a^	< 0.05
Caffeic acid	2.30 ± 0.10^d^	2.80 ± 0.10^c^	3.23 ± 0.15^b^	3.43 ± 0.12^ab^	3.67 ± 0.21^a^	< 0.05
Ferulic acid	4.63 ± 0.15^e^	5.73 ± 0.21^d^	6.30 ± 0.26^c^	7.20 ± 0.10^b^	8.03 ± 0.12^a^	< 0.05
Gallic acid	23.50 ± 1.30^c^	24.27 ± 1.20^c^	25.70 ± 1.20^b^	26.42 ± 2.90^b^	28.70 ± 1.31^a^	< 0.05
Sinapic acid	4.80 ± 0.40^e^	6.60 ± 0.50^d^	8.67 ± 1.10^c^	11.83 ± 1.60^b^	13.46 ± 1.31^a^	< 0.05
Syringic acid	53.83 ± 2.50^d^	59.03 ± 2.70^c^	61.77 ± 2.40^c^	65.07 ± 2.40^a^	66.13 ± 2.65^a^	< 0.05
Phenolic Compounds (Ʃ)	290.23	309.90	324.57	347.37	358.27	

The data presented in the table represent the mean ± standard deviation (n = 3). Values with different superscripts within the same row are statistically significant from each other (*P* < 0.05)

The findings are consistent with the results reported by Al‐Qaisi et al. (2024) and Alrosan et al. (2021). Both studies demonstrated that the presence of LAB during fermentation significantly influences the release of phenolic components. A study conducted by Ripari et al. (2019) revealed that phenolic compounds increased after fermentation by *Lactobacilli* spp. This phenomenon was attributed to the production of phenolic acid esterases by the fermenting bacteria, which transformed plant cell wall polysaccharides into esters. Furthermore, Malka et al. (2023) reported that the TPC of pea seeds increased after fermentation by 1.5 g/100 g. The levels of phenolic compounds in plant-based products are indeed influenced by diverse variables, reflecting the intricate interplay of biological, environmental, and processing factors. These factors include growing conditions, ripening process, processing, storage conditions, genotype, ontogeny, and cultivation techniques (Naczk and Shahidi, 2006). Additionally, the extraction method employed plays a crucial role in determining the phenolic content (Zargoosh et al., 2019).

The levels of phenolic compounds and their presence in various fermentation processes can increase phenolic content. LAB are a group of bacteria that produce lactic acid as a primary metabolic product during the fermentation of carbohydrates (Fu et al., 2024), proteins (Ayala-Niño et al 2019), and fat (Alrosan et al., 2023a). According to Lai et al. (2013), LAB fermentation enhanced the level of phenolic compounds in soy, increasing from 4.60 ± 0.28 to 5.96 ± 0.17 mg GAE/g. A comprehensive analysis of phenolic compounds detected in both fermented and unfermented QPs revealed a diverse range of compounds, including epicatechin, rutin, syringic acid, caffeic acid, sinapic acid, quercetin, gallic acid, catechin, ferulic acid, and chlorogenic acid (Table 3).

The levels of phenolic compounds observed during the fermentation process exhibited an upward trend. Adebo and Gabriela Medina-Meza (2020) reported that fermentation by *Lactobacillus* spp. can augment the levels of quercetin, gallic acid, and catechin during the fermentation. The occurrence of elevated phenolic compounds in fermented proteins is associated with the release of bioactive compounds during the fermentation process. Fermentation, particularly when carried out by microorganisms such as LAB, can induce various biochemical transformations that influence the composition of non-nutritive compounds, including phenolic compounds. It was ascertained that QPs possessed the highest concentrations of epicatechin, chlorogenic acid, and syringic acid (98.63, 53.07, and 53.83 mg/100 g, respectively). Subsequently, after 24 h of fermentation, these amounts experienced a significant (*P* < 0.05) increase and reached approximately 114.10, 63.13, and 66.13 mg/100 g, respectively. In contrast, the initial level of phenolic compounds in QPs (pre-fermentation) was 290.23 mg/100 g. After 24 h of fermentation, the phenolic compound content surged to the highest level observed, amounting to 358.27 mg/100 g.

Phenolic compounds can form complexes with proteins, including glycoproteins, resulting in the insolubility of phenolic compounds in natural systems (Fig. 2). The interactions between phenolic compounds and proteins are diverse, and various factors can influence the solubility of the resulting complexes. Alrosan et al. (2021) demonstrated that phenolic compounds form ester bonds with protein and carbohydrates through hydroxyl and carboxylic groups, respectively. The LAB and their enzymes can break down bonds, releasing smaller protein molecules and water-soluble phenolic compounds. Consequently, it becomes challenging to obtain all the phenolic compounds through a single extraction procedure (Naczk and Shahidi, 2006). Microorganisms release enzymes, such as glycosidases and esterases, capable of cleaving glycosidic or ester bonds in phenolic compounds. This enzymatic activity liberates free phenolic compounds. Fermentation, a biological process employed for centuries in producing diverse food and beverage products, has also expanded its application to include biotechnology for producing biofuels and pharmaceuticals. Furthermore, statistically significant (*P* < 0.05) differences were observed between the TPC and detected phenolic compounds throughout the third and fourth phases of fermentation (Table 3). These variations in phenolic compounds during fermentation suggest that the process plays a pivotal role in releasing and transforming these compounds. Understanding these transformations can aid in optimizing fermentation conditions to maximize the production of desired phenolic compounds for various applications across industries such as food, beverage, biotechnology, and pharmaceuticals.

### Protein–phenolic interactions

The interaction between proteins and phenolic molecules encompasses both covalent and non-covalent interactions (Quan et al., 2019). Covalent interactions, such as forming ester bonds or binding phenolic groups to specific amino acid residues through chemical reactions, contribute to forming protein–phenolic complexes. Non-covalent interactions, including hydrogen bonding, hydrophobic interactions, ionic interactions, and van der Waals forces, also play a role in this process (Quan et al., 2019; Zhang et al., 2021). These interactions establish permanent relationships between particles (Fig. 2), which are typically observed under specific conditions, such as the presence of phenolic chemicals or alkaline environments.

The interaction between phenolic compounds and proteins can have diverse effects on the biological, functional, and nutritional properties of both components. Phenolic compounds are a diverse group of naturally occurring compounds found in plants, renowned for their antioxidant properties. Some phenolic compounds enhance protein digestibility by promoting enzymatic activity. For instance, certain phenolic compounds can serve as cofactors for proteolytic enzymes, facilitating the breakdown of proteins into smaller peptides and amino acids (Alrosan et al., 2024). Phenolics with antioxidant properties may protect proteins from oxidative damage during digestion, preserving their structural integrity and aiding in their breakdown (Adebo and Gabriela Medina-Meza, 2020). Conversely, many phenolic compounds can inhibit protein digestibility. They may form complexes with proteins, reducing their solubility and accessibility to digestive enzymes. Additionally, some phenolics can directly inhibit proteolytic enzymes, hindering the cleavage of protein peptide bonds (Quan et al., 2019). This can result in a decrease in the overall efficiency of protein digestion.

Phenolic compounds, characterized by the presence of hydroxyl functional groups, are known to act as hydrogen donors (Fig. 2). These compounds engage in hydrogen bonding interactions, particularly with protein carboxyl groups. Carboxyl groups in proteins typically possess oxygen atoms with partial negative charges, enabling them to form hydrogen bonds with the hydroxyl groups of phenolic compounds (Alrosan et al., 2021), which exhibit partial positive charges (Fig. 2). Proteins frequently contain amino acid residues with hydroxyl groups, such as serine, threonine, and tyrosine. The oxygen atom in the hydroxyl group of these amino acids participates in hydrogen bonding (Quan et al., 2019). This interaction holds significant implications for proteins’ functional and structural stability.

Phenolic compounds exert inhibitory effects on enzyme activity through various mechanisms, including non-competitive, mixed, allosteric, and competitive inhibitions. The primary mechanism underlying hydrophobic interactions involves the interaction between the hydrophobic regions of protein molecules and non-polar aromatic rings of phenolic compounds (Alrosan et al., 2023b). Additionally, electrostatic interactions occur between the charged particles on protein structures and hydroxyl groups present in phenolic substances.

### Microbial activity

Fermenting microorganisms exhibit significant variations in their growth patterns, influenced by the specific microorganism, fermentation process, and environmental conditions. Fermentation is a metabolic process that transforms sugars into metabolites, such as alcohol or organic acids, utilizing fermenting microorganisms, such as bacteria, yeast, or molds. As presented in Table 1, microorganisms may experience a lag phase during the initial phase characterized by a colony count of 5.92 log CFU/mL. Subsequently, they undergo a dramatic growth surge and proliferation until reaching the final stage of fermentation, approximately 8.94 log CFU/mL. Notably, the growth patterns of distinct fermenting microorganisms exhibit variations throughout the fermentation process, as depicted in Table 1. During the middle fermentation period (12 h), the LAB count surpassed 7.39 log CFU/mL. The growth of colonies is characterized by the replication of individual cells, and the availability of suitable nutrients, such as carbohydrates and proteins, is paramount for this process. Optimal conditions facilitate the efficient utilization of carbohydrates and proteins by microorganisms in a low-acid environment (Liu et al., 2023b).

In conclusion, this study successfully employed *Lactiplantibacillus plantarum* as a fermentation driver to modify QPs. The design of the fermentation process entails optimizing fermentation parameters such as temperature, pH, nutrient availability, and fermentation time to maximize the proliferation and activity of *L. plantarum*. This study investigated the effects of fermentation on the protein structure of QPs and their solubility and digestibility. The fermenting LAB and their enzymes released bound phenolic complexes, leading to the elevation of free phenolic compound levels. Conversely, the quality of QPs exhibited amelioration after 24 h of fermentation utilizing LAB, attributed to modifications to the conformation, secondary, and tertiary structures throughout the fermentation process. Furthermore, the solubility and protein digestibility of QPs increased from 66.86 to 73.05% and from 78.32 to 85.27%, respectively. The significant (*P* < 0.05) increase in digestibility observed in the study underscores the potential of LAB-mediated fermentation as a method to enhance the nutritional quality of QPs. This finding holds implications for fermented quinoa products with improved protein digestibility and overall nutritional value. Moreover, the findings gained from studies like these contribute to the broader comprehension of how fermentation can positively influence the nutritional properties of plant-based protein sources. Future investigations should prioritize exploring fermentation using specific fermenting microorganism species from the water kefir to gain a more comprehensive understanding of the underlying processes that govern the quality and fermentation characteristics of QPs.

## Data Availability

Available on request.
